# Occupational Health Applied Infodemiological Studies of Nutritional Diseases and Disorders: Scoping Review with Meta-Analysis

**DOI:** 10.3390/nu15163575

**Published:** 2023-08-14

**Authors:** Ruben Palomo-Llinares, Julia Sánchez-Tormo, Carmina Wanden-Berghe, Javier Sanz-Valero

**Affiliations:** 1 Department of Public Health and History of Science, School of Medicine, Miguel Hernandez University, 03550 Sant Joan d’Alacant, Spain; palomo.rub@gmail.com; 2 Health and Biomedical Research Institute of Alicante (ISABIAL), Foundation for the Promotion of Health and Biomedical Research in the Valencian Region (FISABIO), 30010 Alicante, Spain; jsancheztorm@gmail.com (J.S.-T.); carminaw@telefonica.net (C.W.-B.); 3 National School of Occupational Medicine, Carlos III Health Institute, 28029 Madrid, Spain

**Keywords:** infodemiology, systematic review, meta-analysis, occupational health, nutrition disorders

## Abstract

(1) Objective: to identify and review existing infodemiological studies on nutritional disorders applied to occupational health and to analyse the effect of the intervention on body mass index (BMI) or alternatively body weight (BW); (2) Methods: This study involved a critical analysis of articles retrieved from MEDLINE (via PubMed), Embase, Cochrane Library, PsycINFO, Scopus, Web of Science, Latin American, and Caribbean Health Sciences Literature (LILACS) and Medicina en Español (MEDES) using the descriptors “Nutrition Disorders, “Occupational Health” and “Infodemiology”, applying the filters “Humans” and “Adult: 19+ years”. The search was conducted on 29 May 2021; (3) Results: a total of 357 references were identified from the bibliographic database searches; after applying the inclusion and exclusion criteria, a total of 11 valid studies were obtained for the review. Interventions could be categorised into (1) interventions related to lifestyle, physical activity, and dietary changes through education programmes, (2) interventions associated with lifestyle, physical activity, and dietary changes through the use of telemonitoring systems or self-help applications, (3) interventions tied to lifestyle, physical activity, and dietary changes through control and/or social network support groups, and (4) interventions linked to changes in the work environment, including behavioural change training and work environment training tasks. The meta-analysis demonstrated that the heterogeneity present when analysing the results for BMI was 72% (*p* < 0.01), which decreased to 0% (*p* = 0.57) when analysing the outcomes for weight, in which case the null hypothesis of homogeneity could be accepted. In all instances, the final summary of the effect was on the decreasing side for both BMI and BW; (4) Conclusions: Despite the high heterogeneity of the results reported, the trend shown in all cases indicates that the intervention methodologies implemented by empowering individuals through Web 2.0 technologies are positive in terms of the problem of overweight. Further implementation of novel strategies to support individuals is needed to overcome obesity, and, at least in the early studies, these strategies seem to be making the necessary change.

## 1. Introduction

The importance of physical activity and proper nutrition for health is often discussed. Meanwhile, health and work often go hand in hand and have an impact on each other. In this pairing, it should be borne in mind that many people take at least one of their other daily meals at the workplace, making nutrition an essential part of working life [1].

In this regard, the National Institute for Occupational Safety and Health (NIOSH) promoted the creation of safe and healthy workplaces within the “Total Worker Health (TWH)” programme, a strategy that integrates occupational safety and health protection to forestall worker injuries and illnesses and improve their health and well-being, with access to healthy and affordable food being an important issue [2]. By the same token, the World Health Organisation’s document on “The workplace as a setting for interventions to improve diet and promote physical activity” demonstrated that the workplace provided a great opportunity to improve health, as it was a centre where information and training in nutrition and healthy eating could be fostered among workers [3]. The International Labour Organisation, for its part, has found that proper nutrition can help prevent chronic diseases, lessen workplace accidents and sick days, and protect against pollution and chemical agents in the working environment [4].

Despite the high incidence of obesity and its related diseases, obesity mitigation strategies have not proven effective. Over the past 30 years, no World Health Organisation member country has been able to reverse the trend of increasing obesity in the population. Therefore, it has been considered essential to use new approaches, some of them based on 2.0, to try to reverse this situation [5].

Although Occupational Health is the advancement and upkeep of the most astounding level of physical, mental, and social health of specialists in all occupations by preventing departures from well-being, controlling dangers, and the adjustment of work to individuals and individuals to their jobs. Nutrition and occupational health are the maintenance and promotion of the highest degree of physical, mental, and social health of workers in all occupations by controlling risks, promoting healthy eating, providing humanitarian aid, improving health systems, and preventing departures from health [6].

In this context, it is important to note that nutrition disorders are diseases that occur when a person’s dietary intake does not contain the right amount of nutrients for healthy functioning, or when a person cannot correctly absorb nutrients from food. Nutritional disorders include a wide spectrum of conditions, including generalised undernutrition, overnutrition leading to obesity, eating disorders, and diseases where nutrition has a role in the aetiology [7].

Nutritional disorders have contributed substantially to a variety of chronic diseases, including diabetes, cardiovascular diseases, hyperlipidaemia, and arthritis. Thus, it is recognised that morbidity related to overnutrition gives rise to indirect labour costs in terms of productivity loss due to both presenteeism and absenteeism. Understanding the factors tied to obesity in the workforce is therefore essential to developing effective interventions [8].

Amid current trends focusing on nutrition and occupational health, the creation of a new concept, nutra-ergonomics, stands out. It is defined as “the interface between workers, their work environment, and their performance in relation to their nutritional status. Nutrition is seen as an integral part of a safe and productive workplace, encompassing the physical and mental health and long-term well-being of workers” [9,10].

Additionally, it is well known that the dawn of Web 2.0 resources has provoked a substantive change in the communication of knowledge, favouring its disclosure by enabling the expansion and permeability of knowledge at a very low cost. Web 2.0 has shown its integration in today’s information society and, far from dwindling, increasingly has more initiatives that enhance it, subsequently contributing to the diffusion of the content about health [11].

Nowadays, the increasing use of social networking and interactive resources is undeniable. Over the last decade, the use of web-based data on public health issues has proved to be handy in understanding the behaviour of people seeking this information [12]. While traditional methods could only aim to understand the reasons or situations that led individuals to take their actions, the new methods related to data science can perform newcasting tasks (knowledge of the present), i.e., predicting values that are befalling at the same time as the data is generated, and can also be used in forecasting tasks (prediction of future trends) [13]. This is achieved either by machine learning techniques [14] or by infodemiological techniques [15,16].

In the health field, Eysenbach [15,16] coined the term “infodemiology” (information + epidemiology) as an emerging set of public health information methods in order to analyse search behaviour, communication, and publication on the Internet. That is, observing and analysing the behaviour based on the Web to know the human demeanour with the purpose of foretelling, assessing, and even preventing the health-related problems that constantly arise in quotidian life [17].

The field of infodemiology has been demonstrated to be particularly useful in the early detection of patient health events, from the early onset of epidemics, such as diabetes [18], obesity [19], or occupational health [20]. For instance, MacKinlay et al. [21] showed that the use of information technologies applied to social networks decreased the time to access information when an adverse drug event occurred.

In the field of occupational health, these new digital tools applied to the epidemiological surveillance of occupational diseases could be very useful. Complete follow-up using new techniques may be essential for the surveillance of occupational health conditions [20].

Given these technologies, attempts are being made to create apps that promote physical activity and healthy diets. Nevertheless, they are not achieving long-term user loyalty. This may be due to two factors. On the one hand, those more generalist applications that are present in most digital markets do not have sufficient scientific advice, and on the other hand, those initiatives that have advice generally do not have the financial and marketing resources to achieve the desired success. It would be interesting to have platforms that combine both factors and try to generate a higher rate of user follow-up [22].

Thus, it would be interesting to know which digital tools that contribute to the discovery of the most sought-after services, such as what are the new trends and the needs demanded by users, and whether they are applicable to the improvement of workers ‘health. For this reason, the objective of this study was to find out about and review existing infodemiological studies on nutritional disorders applied to occupational health.

## 2. Materials and Methods

### 2.1. Design

A scoping review of the papers was retrieved using the systematic technique. The extension for exploratory reviews suggested by the PRISMA (Preferred Reporting Items for Systematic Reviews and Meta-Analyses) statement was followed [23].

### 2.2. Data Source

Data were procured from direct consultation and access, via the Internet, to the following bibliographic databases in the field of health sciences: MEDLINE (via PubMed), Embase, Cochrane Library, Scopus, Web of Science, Latin American, and Caribbean Literatures in Health Sciences (LILACS) and Medicina en Español (MEDES).

### 2.3. Information Processing

To define the search terms, we used the Thesaurus of Health Sciences Descriptors (DeCS), developed by the Latin American and Caribbean Centre for Medical Sciences Information (BIREME), and its equivalent, the Medical Subject Headings (MeSH), developed by the US National Library of Medicine.

The following search equations were considered appropriate after studying the hierarchy of both the thesaurus and their index cards (entry terms):

Equation 1: Nutrition Disorders: Disorders caused by nutritional imbalance, either overnutrition or undernutrition.

“Nutrition Disorders”[Mesh] OR “Nutrition Disorder*”[Title/Abstract] OR “Nutritional Disorder*”[Title/Abstract] OR “Deficiency Disease*”[Title/Abstract] OR “Avitaminosis”[Title/Abstract] OR “Malnutrition”[Title/Abstract] OR “Starvation”[Title/Abstract] OR “Magnesium Deficiency”[Title/Abstract] OR “Potassium Deficiency”[Title/Abstract] OR “Protein Deficiency”[Title/Abstract] OR “Overnutrition”[Title/Abstract] OR “Overweight”[Title/Abstract] OR “Obesity”[Title/Abstract] OR “Wasting Syndrome”[Title/Abstract] OR “Feeding Disorder*”[Title/Abstract] OR “Food Allergy”[Title/Abstract] OR “Nutritional Deficiency”[Title/Abstract] OR “Nutritional Intolerance”[Title/Abstract] OR “Refeeding Syndrome”[Title/Abstract].

Equation 2: Occupational Health: The promotion and maintenance of physical and mental health in the work environment.

“Occupational Health”[Mesh] OR “Occupational Health”[Title/Abstract] OR “Industrial Hygiene”[Title/Abstract] OR “Industrial Health”[Title/Abstract] OR “Occupational Safety”[Title/Abstract] OR “Employee Health”[Title/Abstract] OR “Occupational Risk*”[Title/Abstract] OR “Insecure Labor Condition*”[Title/Abstract] OR “Work Risk*”[Title/Abstract] OR “Occupational Hazard*”[Title/Abstract] OR “Risk at Work”[Title/Abstract] OR “Professional Health”[Title/Abstract] OR “Working Condition*”[Title/Abstract] OR “Occupational Stress”[Mesh] OR “Job Stress”[Title/Abstract] OR “Professional Stress”[Title/Abstract] OR “Work Place Stress”[Title/Abstract] OR “Workplace Stress”[Title/Abstract] OR “Work Medicine”[Title/Abstract] OR “Work”[Title/Abstract].

Equation 3: Infodemiology: The science of distribution and determinants of information in an electronic medium, specifically the Internet, or in a population, with the ultimate aim to inform public health and public policy.

“Infodemiolog*”[Title/Abstract] OR “Infoepidemiolog*”[Title/Abstract] OR “Info Epidemiolog*”[Title/Abstract] OR “Infoveillance”[Title/Abstract] OR “Infosurveillance”[Title/Abstract] OR “Info Surveillance”[Title/Abstract] OR “Google Trend*”[Title/Abstract] OR “Google Analysis”[Title/Abstract] OR “Internet Trend*”[Title/Abstract] OR “Information Trend*”[Title/Abstract] OR “Network Trend*”[Title/Abstract] OR “Online Search*”[Title/Abstract] OR “Online Trend*”[Title/Abstract] OR “Infodemic”[Title/Abstract] OR “Googling”[Title/Abstract] OR “Social Networking”[Mesh] OR “Social Media”[Title/Abstract] OR “Blog”[Title/Abstract] OR “Wikipedia”[Title/Abstract] OR “Wiki”[Title/Abstract] OR “YouTube”[Title/Abstract] OR “Facebook”[Title/Abstract] OR “Twitter”[Title/Abstract] OR “Tiktok”[Title/Abstract] OR “Tik-tok”[Title/Abstract] OR “Sina Weibo”[Title/Abstract] OR “SinaWeibo”[Title/Abstract].

The final search equation was developed for use in the MEDLINE database, via PubMed, by the Boolean Union of the three suggested equations: Equation 1 AND Equation 2 AND Equation 3, with the filters: Humans: “Humans” and adults “Adult: 19+ years”. Subsequently, this strategy was adjusted to the characteristics of each of the other consulted databases, with the search running from the first available date in each of the selected databases until February 2023.

In addition, to lessen the possibility of publication bias, a supplementary search was conducted by manually searching the reference lists of the articles selected for the review and related systematic reviews. The list of similar articles provided by MEDLINE for each of the selected trials was also checked.

### 2.4. Final Selection of Articles

Articles that met the following criteria were selected for review and critical analysis:

- Inclusion: clinical trials published in peer-reviewed journals and written in English, Spanish, and Portuguese;
- Exclusion: articles for which the full text could not be found, those that included a non-adult population (under 18 years of age), and those that did not present a relationship between the intervention and the outcome under study (causality criterion). Interventions had to focus on the field of infodemiology and include a Web 2.0 tool. This intervention should have been carried out in the workplace to improve/detect/plan problems related to nutritional diseases or nutritional disorders. The outcome had to be measurable and preferably facilitate differences in body mass index or its effect on body weight.

Adequacy of the selection of these articles was carried out by the authors of this review. To validate the inclusion of articles, it was specified that the concordance assessment of the selection (kappa index) had to be greater than 0.60 [24]. Provided this condition was met, any discrepancies were resolved by consensus of all review authors.

### 2.5. Documentary Quality, Level of Evidence and Recommendation, and Study of Bias

The adequacy of the selected articles was evaluated through the CONSORT (CONsolidated Standards of Reporting Trials) guidelines for reporting observational studies [25], which contain a list of 25 essential checkpoints that should be described in the publication of these papers. For each selected article, one point was assigned for each item present (if not applicable, not scored). When an item consisted of several sections, these were scored independently, with each section receiving the same score, and then averaged (being this the final score of that item) so that in no case did the total score exceed one point per item. Item 19 has not been considered in the assessment as there were no adverse effects caused by this type of intervention.

The recommendations of the Scottish Intercollegiate Guidelines Network (SIGN) Grading Review Group [26] were used to determine the level of evidence and the level of recommendation.

The RoB.2 clinical trial bias estimation tool [27,28] was utilised to evaluate the possible biases of the trials included in the review. Bias was assessed using the criteria of high, low, or doubtful bias for the dimensions: D1 Bias arising from the randomisation process, D2 Bias due to deviations from intended intervention, D3 Bias due to missing outcome data, D4 Bias in the measurement of the outcome and D5 Bias in the selection of the reported result.

The study of publication bias was performed using the funnel plot [29].

### 2.6. Data Extraction

The articles were grouped according to the variables under study to systematise and simplify the understanding of the results, considering the following data: first author and the year of publication; the population demographics: the size of the sample, age, and gender composition and the country they are from; the intervention data; the reported pathology; the Social Platform that was used during the intervention and the compilation time; and the results: the measured outcomes (weight loss or BMI increase or both if applicable) and the significance of the statistical test used to compare the outcomes for Table 1. Moreover, the first author and the year of publication, a description of the intervention, a description of the results, and a description of the conclusions were included in Table 2.

The control of the data correction was conducted through double tables, which allowed the detection of divergences and their revision by reconsulting the originals.

The removal of duplicate records (present in more than one database) was conducted using the multi-platform programme ZOTERO (bibliographic reference manager developed by the Centre for History and New Media at George Mason University).

The Burton–Kebler (BK) and Prince Index (PI) were calculated to determine the timeliness of the studies.

### 2.7. Goal of the Meta-Analysis

Meta-analysis provides a more precise estimate of the effect size and increases the generalizability of the results of individual studies. The larger sample sizes can provide trends and yield conclusive results when individual studies are inconclusive; it may enable the resolution of conflicts between studies, and influence future research. In this sense, and given the possible heterogeneity in the applied methodologies, the objectives of the meta-analysis carried out were:

- Determine the effectiveness of the methodologies applied in the different studies;
- Study the significant results for the outcomes of Body Weight and Body Mass Index;
- Observe the general trends, if any, of the results presented.

### 2.8. Data Analysis

Data related to information retrieval were presented in terms of frequency and percentage.

To ascertain the BK, the median age was calculated according to the analysed time, and the PI was computed as the percentage of articles with an age of fewer than 5 years.

The measure of concordance to ascertain the adequacy of the selection of articles was conducted using the IK. The relationship between authors was considered valid when its value was greater than 60% (good or very good concordance strength).

The CONSORT questionnaire scores were studied using the median, maximum, and minimum scores. The evolution of this score in relation to the years of publication was procured through Pearson´s correlation analysis.

The Kruskal–Wallis test was used to compute the relationship between the differences in BMI and BW amid the different platforms used and the interventions performed.

For the meta-analysis, it has been employed the standardised mean effects technique with Hedges’ g and the Knapp-Hartung adjustment. In addition, inter-study variability estimated with the variance amid them τ^2^ and its statistical significance through Wald’s Q were used to study heterogeneity.

Data analysis was performed using R v4.2.2 software with RStudio 2022.12.0 build 353 work package. The specific library used to calculate the risk of bias was “robvis” v0.3.0.900, while the specific library utilised for analysis was “meta” v6.1-0.

### 2.9. Ethical Aspects

All data were procured from articles accepted for review. Hence, and in accordance with 14/2007 on biomedical research [30], approval from the Ethics and Research Committee was not required when using secondary data.

**Table 1:** Summary of the accepted articles for review on occupational health infodemiological studies on nutritional diseases and disorders.

	Population				Intervention			Results	
	Size	Age (Years)	Gender (% M/F/O)	Country	Disorder/Disease	Platform	Compilation Dates	Outcomes	*p*-Value
Hene et al., 2022 [31]	N = 300 ni = 100	>18	Not reported	South Africa	Cardiovascular Disease	Facebook	12 months	ΔBW = −1.5	<0.01
Kariuki et al., 2022 [32]	N = 82 ni = 41	>18	Not reported	USA	Overweight	YouTube	6 months	ΔBW = −5.6 ΔBMI = −1.8	<0.05
Napolitano et al., 2022 [33]	N = 456 ni = 304	18–35	Not reported	USA	Overweight	Facebook	55 months	ΔBMI = −3.6	<0.05
Moholdt et al., 2021 [34]	N = 24 ni = 16	30–40	100/0/0	Australia	Overweight	AUC Web	6 months	ΔBW = −6.7 ΔBMI = −0.75	<0.05
Biederman et al., 2021 [35]	N = 27 ni = 20	>18	0/100/0	USA	Overweight	Facebook	1.25 months	ΔBW = −1.4 ΔBMI = −0.9	<0.05
Xu et al., 2021 [36]	N = 47 ni = 39	35–65	4/94/2	USA	Overweight	Facebook	3 months	ΔBW = −1.25	<0.05
Osborn et al., 2020 [37]	N = 99 ni = 49	>18	27/73/0	USA	Diabetes Mellitus	One Drop App	6 months	ΔBMI = −1.0	<0.001
Hawkins et al., 2020 [38]	N = 369 ni = 299	>18	13/87/0	Finland	Nutritional Disorder	Facebook	10 months	ΔBMI = −0.6	<0.01
Dagan et al. 2015 [39]	N = 63 ni = 30	>18	30/70/0	USA	Dietary Habits	Facebook	3 months	ΔBMI = −1.2	0.25
Huei Phing et al., 2015 [40]	N = 120 ni = 60	>18	18/82/0	Malaysia	Metabolic Syndrome	Facebook	4 months	ΔBW = −7.0 ΔBMI = −3.4	<0.001
Merchant et al., 2014 [41]	N = 404 ni = 202	18–35	Not reported	USA	Overweight	Facebook	12 months	ΔBMI = −0.3	<0.001

N: Total population size; ni: the size of the intervention group; % M/F/O: Percentage of males, females, and others; ΔBMI: Increment of Body Mass Index, ΔBW: Increment of Body Weight; One Drop: A free app for people with diabetes; AUC Web: Australian University Catholic Web.

**Table 2:** Summary of the intervention for the accepted articles for review.

	Intervention	Results	Conclusion
Hene et al., 2022 [31]	Financial sector employees were randomly assigned to three intervention groups (Facebook plus Health Professionals, Facebook only groups, and control) to reduce 10-year cardiovascular disease risk (Framingham risk score FRS).	Overweight and diabetes risk reduced significantly in Facebooks groups versus control.	Social network lifestyles could be included in workplace health promotional programs to improve certain non-communicable disease risk factors.
Kariuki et al., 2022 [32]	12-week follow-up randomised controlled trial with control and intervention (YouTube and FitBit remote coach) groups to increase physical activity (PA).	Increase of PA time of the intervention group (89.5%). The intervention group shows improvement in weight, BMI, body fat, waist circumference, and systolic BP.	This trial demonstrated that the intervention is feasible and acceptable and provided preliminary efficacy in promoting PA among adults with overweight/obesity.
Napolitano et al., 2022 [33]	A randomised trial of university students to translate and deliver programs via social media (Facebook) of social media treatments, Social Support, and Daily text messages. Eighteen months of individual follow-up to improve the quality of life of the overweight population	6- and 18-month BMI significantly decrease in the intervention group versus the control group.	The results of this study have the potential to significantly impact the delivery of obesity treatment services on Collages Campuses.
Moholdt et al., 2021 [34]	Australian male university workers with overweight problems are enrolled in a three-arm trial. The goal is to monitor the effects of morning vs. evening training vs. no training and monitor with social media University website.	Both training groups show BMI and weight loss against the no-training group. There is no significance in these parameters between the morning and evening groups.	Improvements in cardiorespiratory fitness were similar regardless of the time of day of exercise training.
Biederman et al., 2021 [35]	A randomised trial of 5-week physical activity (PA) program for African American women of working age using intervention Facebook groups. Intervention groups were monitored with an Omron Alvita pedometer to monitor the daily steps and number of days they were physically active.	The intervention group had significantly increased their weekly steps by 190% as compared to the control group. The intervention group showed a decrease in both BMI and Body Weight.	Technologies such as social media and pedometers can assist in educating individuals and improving physical activity. These findings are relevant to public health nurses when implementing programs to increase physical activity for African American women.
Xu et al., 2021 [36]	A clinical trial of a 12-week intervention period with a dietary and physical activity intervention group of participants via Facebook. Performed mediation analyses to explore how the effects of social network measures on weight loss could be mediated by the theoretical mediators.	Increases in the number of posts, comments, and reactions significantly predicted weight loss. Receiving comments positively predicted changes in self-efficacy. Active participants show significant Body Weight loss over baseline.	The potential of using social network analysis to understand the social processes and mechanisms through which web-based behavioural interventions affect participants’ psychological and behavioural outcomes.
Osborn et al., 2020 [37]	Social media were used to recruit T1 Diabetes adults. Two groups were generated, one with the One-Drop activity tracker and the other without any activity tracker. Three-month follow-up for the activity modification on the T1D behaviour.	Participants in the One Drop activity tracker condition had a significantly lower 3-month haemoglobin A_1c_ level. It also shows a significant decrease in BMI levels for the intervention group.	Participants exposed to the One Drop activity tracker had a significantly lower 3-month haemoglobin A_1c_ level compared to that of participants not exposed to One Drop during the same timeframe.
Hawkins et al., 2020 [38]	This study examined whether four perceived norms (perceived descriptive, injunctive, liking, and frequency norms) about Facebook users’ eating habits and preferences predicted participants’ own food consumption and BMI. The four users’ consumptions were fruit, vegetables, energy-dense snacks, and sugar-sweetened beverages. The study aims to change consumption behaviour with interventions on the perceived norms of two advertising groups.	When the Facebook users were shown the different perceived norms, their eating behaviour changed significantly. In that regard, there was a significant decrease in the BMI of the participants of the intervention group.	These findings suggest that perceived norms concerning actual consumption (descriptive and frequency) and norms related to approval (injunctive) may guide the consumption of low and high-energy-dense foods and beverages differently.
Dagan et al., 2015 [39]	As an intervention, it was developed Food Hero, an online platform within Facebook for nutritional education in which players feed a virtual character according to their own nutritional needs and complete a set of virtual sports challenges. The platform was developed in 2 versions: a “private version”, in which a user can see only his or her own score, and a “social version”, in which a user can see other players’ scores, including preexisting Facebook friends.	Users that have the “social version” tend to behave more responsibly that those with the “private version”. In spite of that, the BMI decrease of the “public group” was not significantly lower than the “private group”.	This work focused on isolating the social networks’ social effects to help guide future online interventions. Our results indicate that the social exposure provided by SNSs is associated with increased engagement and learning in an online nutritional educational platform.
Huei Phing et al., 2015 [40]	The purpose is to ascertain the effect of a physical activity intervention using a combination of Facebook and standing banners on improvements in metabolic syndrome. Government employees with metabolic syndrome were randomly placed in a two-arm trial. A Lifecorder e-STEP accelerometer was utilised to quantify physical activity.	There were significantly higher step counts in the intervention group as compared to the control group over time. Both the Body Weight and the BMI of the intervention group improved over the control group.	The findings show that delivering information on physical activity through an easily implemented and low-cost physical activity intervention via a combination of Facebook and standing banners was successful in improving step counts and metabolic parameters among individuals with metabolic syndrome.
Merchant et al., 2014 [41]	The basis of the intervention campaign model was five self-regulatory techniques: intention formation, action planning, feedback, goal review, and self-monitoring. Participants were encouraged to engage their existing social network to meet their weight loss goals. A health coach moderated the page and modified content based on usage patterns and user feedback.	There was significant variability among quantifiable (i.e., visible) engagement. Approximately 40% of the participants interviewed reported passively engaging with the Facebook posts by reading but not visibly interacting with them. The more engaged users showed a significantly lower BMI than those less engaged in the activity.	Facebook can be used to remotely deliver weight loss intervention content with the help of a health coach who can iteratively tailor content and interact with participants.

## 3. Results

A total of 357 references were retrieved using the search criteria: 69 (19.3%) in MEDLINE (via PubMed), 41 (14.5%) in Embase, 71 (19.9%) in Cochrane Library, 73 (20.5%) in Scopus, 99 (27.7%) in Web of Science and 4 (1.1%) in LILACS. No documents were retrieved from the MEDES bibliographic database. No new documents were found by consulting the bibliographic lists of the selected articles.

**Figure 1:**
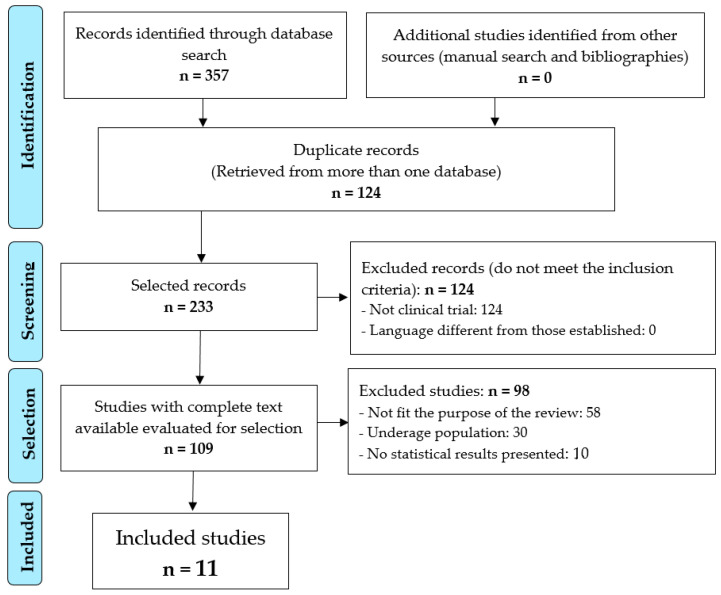
Identification and selection of studies.

After screening the 124 repeated records and applying the inclusion and exclusion criteria (Figure 1), 11 papers [31,32,33,34,35,36,37,38,39,40,41] were selected for review; see Table 1 for the outcomes and Table 2 for the interventions performed.

The agreement between the reviewers on the appropriateness of the selected studies, calculated using the Kappa Index, was 100.0% (*p* < 0.01).

The selected articles had an obsolescence of 2 years, according to the Burton-Kebler Index, with a Price Index of 72.7%. The years with the highest number of published articles were 2022 and 2021, each with three studies.

When assessing the appropriateness of the studies via the CONSORT verification guideline, the percentages of compliance ranged from a minimum of 47.9% to a maximum of 100.0%, with a median of 72.9%. A low, non-significant direct linear trend was observed (R^2^ = 0.07; *p* = 0.44); see Table 3.

The study with the largest population belonged to Napolitano et al. [33], with N = 456 people (304 in the intervention group), and the study with the smallest population was based on Moholdt et al. [34], with N = 24 people (16 in the intervention group). This population was predominantly female, except in the study by Moholdt et al. [34], where 100% of the participants were male. In 4 (36.4%) of the 11 studies [31,32,33,41], the gender of the population was not reported.

The United Stated was the largest contributor, with seven papers (63.6%) [32,33,35,36,37,39,41]. South Africa [31], Australia [34], Finland [38], and Malaysia [40] submitted 1 (9.1%) paper.

### 3.1. Bias Study

**Figure 2:**
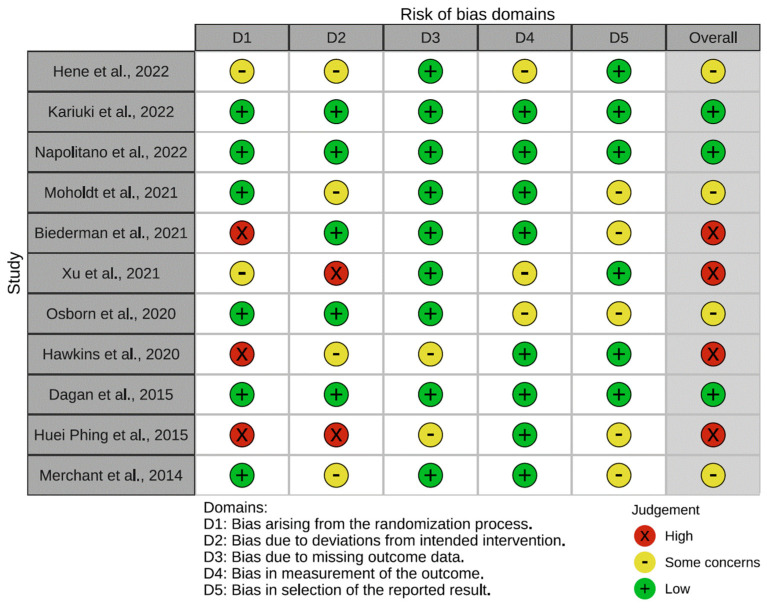
Assessing the methodological risk of clinical trials reviewed through the RoB.2 tool [31,32,33,34,35,36,37,38,39,40,41].

The biases of the trials included in the review, as assessed by the RoB.2 tool, which evaluates the methodological risk of bias, are demonstrated in Figure 2. In view of this graphic, it can be seen that the majority of the bias introduced in the reviewed clinical trials stemmed from the process of patient selection and randomisation of participant groups. By the same token, the process of dealing with missing data, the statistical treatment of the data, and the reporting of the data were not considered to be a determining factor when it comes to the evaluation of bias in the analysed articles.

**Figure 3:**
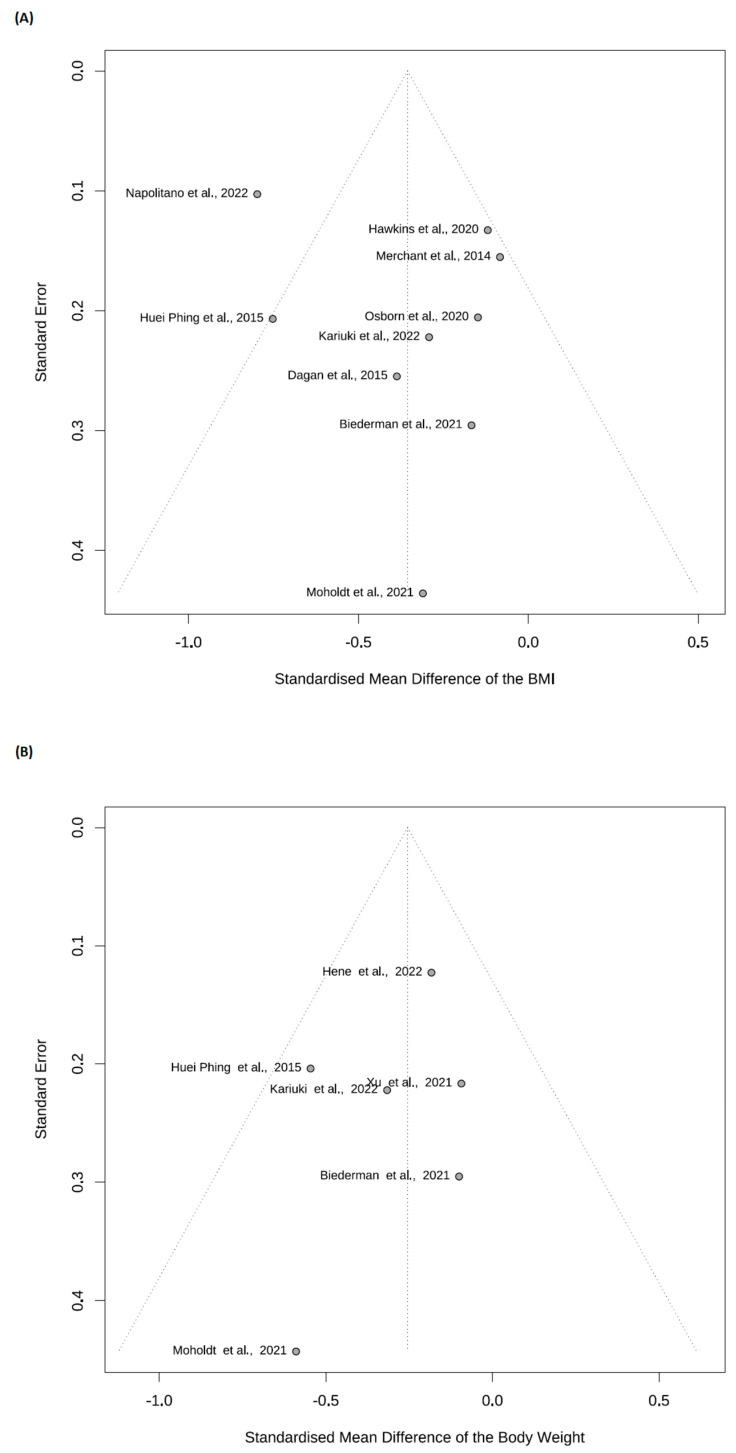
Funnel plot for the study of publication bias [31,32,33,34,35,36,37,38,39,40,41]. (**A**) Body Mass Index; (**B**) Body Weight.

The funnel plot shows that publication bias was not particularly noticeable for body weight, nor for body mass index, see Figure 3.

Thus, based on the SIGN criteria, this review provided evidence with a grade of 1+ (systematic review of randomised clinical trials or randomised clinical trials with low risk of bias) with a grade of recommendation A (a body of evidence consisting mainly of studies rated 1+ that are directly applicable to the target population and show overall consistency of results).

### 3.2. Interventions Performed in the Reviewed Trials

The main disorder/disease studied was Overweight in 6 (54.5%) of the 11 trials [32,33,34,35,36,41]. The study carried out by Hene et al. [31] examined cardiovascular disease; Osborne et al. [37] focused on diabetes mellitus; Hawkins et al. [38] based their study on a population with nutritional disorders; Dagan et al. [39] analysed the effect of the intervention on Dietary Habits and finally Huei Phing et al. [40] selected participants with metabolic syndrome.

The platform used to perform the intervention was mostly Facebook in 8 (72.7%) of the 11 reviewed trials [31,33,35,36,38,39,40,41]. Other applications used only once were YouTube [32], AUC Web [34], and One Drop App [37].

Data collection periods ranged from a maximum of 55 months [33] to a minimum of 1.25 months [35]. Except for the latter trial [35], all the other studies used periods of 3 or more months.

According to the delivered interventions, the reviewed trials could be grouped into four different types:

1. Interventions related to changes in lifestyle, physical activity, and eating behaviour through educational programmes: 2 trials [33,39];
2. Interventions involving changes in lifestyle, physical activity, and eating behaviour using telemonitoring systems or self-help applications: 2 studies [35,37];
3. Interventions tied to changes in lifestyle, physical activity, and eating behaviour through control and/or social network support groups: 4 trials [32,34,38,41];
4. Interventions related to change in the work environment, including behavioural change and fields of work training: 3 trials [31,36,40].

### 3.3. Results Obtained from the Interventions Implemented

Nine (81.8%) of the eleven analysed clinical trials [32,33,34,35,37,38,39,40,41] examined the change in BMI and found a significant decrease in BMI, except for the trial performed by Dagan et al. [39], in which there was a lessen in BMI, but it was not significant. In addition, six studies [31,32,34,35,36,40] examined the change in BW, showing that there was a significant diminish in weight. It should be noted that four trials [32,34,35,40] analysed either the change in BMI or the variation in BW, with significant losses in both cases.

The largest diminish in BMI was found in the study carried out by Napolitano et al. [33], with a value of −3.6 kg/m^2^. For BW, the largest reduction was verified in the study by Huei Phing et al. [40], with a loss of 7 kg. In both cases, the intervention was performed via Facebook.

The variation in body mass index and body weight in the reviewed trials is shown in Table 1.

The use of Facebook resulted in a mean loss of BMI of −1.7 ± 0.6 kg/m^2^ and BW of −2.8 ± 1.4 kg in 8 (72.7%) of the 11 reviewed studies [31,33,35,36,38,39,40,41]. The trial that used YouTube [32] demonstrated a lessen in BMI of −1.8 kg/m^2^ and BW of −5.6 kg. Use of the AUC Web [34] resulted in a decrease either in BMI of −0.8 kg/m^2^ or in BW of −6.7 kg. Finally, the study that used the One Drop app [37] as an intervention showed a diminish in BMI of −1.0 kg/m^2^.

According to the platform used, the relationship between the differences in BMI and BW showed no significant differences for either BMI (*p* = 0.79) or BW (*p* = 0.61). There were also no meaningful differences amid the four interventions observed: BMI (*p* = 0.21) and BW (*p* = 0.54).

**Figure 4:**
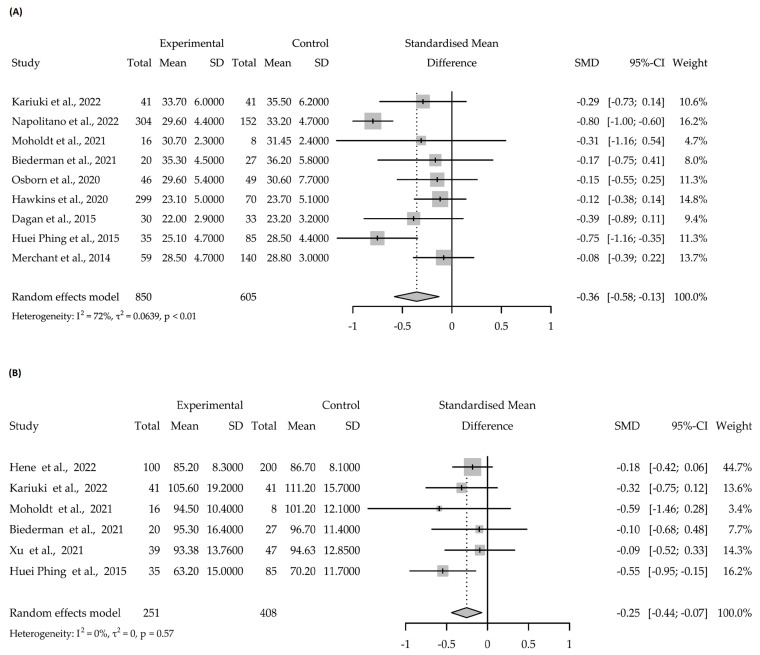
Forest plot of the reviewed clinical trials [31,32,33,34,35,36,37,38,39,40,41]. (**A**) Body Mass Index; (**B**) Body Weight.

The meta-analysis performed demonstrated heterogeneity of 72% (*p* < 0.01) when analysing the results for body mass index, which diminished to 0% (*p* = 0.57) when examining the outcomes for body weight, where the null hypothesis of homogeneity might be accepted. In both cases, the final summary of the effect was on the decreasing side for both BMI and BW. The effect sizes calculated from the meta-analysis are shown in Figure 4.

## 4. Discussion

This study is part of a growing body of literature that has been called “infodemiology”. This includes analysis of search engine queries (the “demand” side), but also what is being published on websites, blogs, etc (the “supply” side) [15].

According to the recommendations on the objectives of a systematic review [42], this meta-analysis review synthesised and analysed the evince related to infodemiological studies applied to occupational health on nutritional diseases and disorders, with the aim of providing relevant information that can help to foster new interventions for the protection of workers [1]. Thus, it can be concluded that the final summary of the effect of both BMI and BW indicates their decrease and, hence, the need to rely on infodemiological studies when designing strategies for the prevention of obesity and overweight in workers.

In addition, this study has accounted for the World Health Organization’s strategy, which underlines the importance of establishing primary prevention and interventions to enhance occupational health [43].

The articles reviewed had very low obsolescence, indicating that this is an emerging topic. In fact, the first article describing infodemiology was published by Eysenbach in December 2002 [44]. The use of Web 2.0 technology is also new: although it was first used in 1999, it was not until the O’Reilly and Dougherty conference in 2004 that its use and development became popular [45]. It is, therefore, logical that the use of these technologies applied to occupational health is highly topical and has much lower obsolescence data than those found in previous reviews related to nutrition and public health [1,46].

Using the CONSORT criteria, the assessment of document adequacy was consistently in line with other similar systematic reviews [47,48] or even found to be slightly higher on average. These findings support the previous paragraph, as the more recent the articles, the greater the degree of homogeneity and compliance with CONSORT criteria in the articles analysed. In this context, Turner et al. [49] demonstrated that the adoption of these criteria improved the quality of the articles. The study of the non-significant time trend in document adequacy was inconsistent with the expected trend of increasing adequacy over time [50]. In this review, given that all the articles were published well after the publication of the first article that used the CONSORT criteria in 1996 [25], the trend line of the assessment was almost horizontal. This homogeneity in the results when analysing the different articles could indicate an adequate application of the criteria in most of the reviewed articles.

The duration of data collection was in the expected range in most cases (3 to 12 months). Only one paper [35] reported a data collection period shorted than recommended (5 weeks). In occupational health, periods of several weeks or even months are considered crucial to evaluate the effects of the interventions [51,52]. Interventions related to being overweight and obese are often successful in the short term, but maintaining the effect in the long term is much more difficult and requires specific follow-up to enhance healthy behaviours [53].

In the gender distribution, except for articles whose inclusion criteria were “men only” [34] or “women only” [35], there was a clear predominance of women in the distributions. Although the female population in the health sector is larger than the male population (women represented 70% of the health and social work workforce) [54], the scientific literature is deficient in a real analysis of women’s participation in full-time employment (excluding the role of full-time caregivers without job recognition) [55]. By the same token, one-third of the reviewed articles did not report data disaggregated by gender, because user behaviour was analysed anonymously in the comments section of social networks, which did not allow researchers to know the gender of the users being evaluated. All these circumstances have hampered a more rigorous analysis of sex/gender considerations in the review process, aiming to ensure health equity for all [56].

Approximately two-thirds of the analysed articles were produced in the USA. This could be attributed to three factors: Firstly, scientific production is directly linked to the quality of the universities of origin, and US universities are always at the top of the excellence rankings [57]. In addition to the reputation of their universities, the significant public and private funding of their institutions and research centres contributes to this [52]. Secondly, it was found that most of the analysed studies focused mainly on the study of overweight in the general and working population. It is well known that this is particularly important in the USA [58,59], and therefore it is logical that many of the study articles are concerned with this issue. Finally, English is the dominant language in the scientific field, so English-speaking countries will find it easier to publish their work [60].

### 4.1. Bias Study

In many clinical trials, the highest biases occur in areas related to statistical calculations, and the lowest biases occur in areas associated with selection and randomisation [1]. In contrast, this review found a different result. When analysing trials in which the data collection was performed through social networks and was, therefore, less controlled by the researchers, this was the biggest source of bias. This means that no bias was found in this area, as one of the selection criteria for this review was ‘the presence of causal results. Finally, the funnel plot showed no major publication bias.

The level of evidence and recommendations found in this review, from randomised clinical trials with a low risk of bias, provided a consistent body of evidence that was directly applicable to the target population.

### 4.2. Interventions in the Reviewed Trials

The interventions observed in the reviewed trials are consistent with the TWH criteria for achieving protection against work-related health and safety risks with the promotion of injury and illness prevention efforts to improve the well-being of workers. TWH by integrating the traditional focus on work-specific factors with attention to health conditions and the quality of working life, the TWH approach provides a pathway to improve worker creativity, innovation, and productivity by creating work and work environments that are safe, health-enhancing, meaningful, and fulfilling [2]. However, Web 2.0-based nutritional interventions to improve workers’ health and performance are scarce. Importantly, nutrition is an essential part of economic development because it influences the health and productivity of workers.

Most studies used the Facebook platform, a tool that has been shown to be effective in recruiting individuals for clinical trials [61], and in all cases, shifted over time away from platforms with more ephemeral content, such as TikTok or Twitter, which currently only have potential benefits in terms of unidirectional information transfer [62,63]. A few of them use a proprietary smartphone app for promoting physical activity with significant results, contrary to what was reported by Arigo et al. [22].

The presence of Facebook in most of the reviewed studies was expected; this Web 2.0 tool has been placed among the three most commonly used in the world and has already shown its potential for health promotion. As the Centres for Disease Control and Prevention indicates, Facebook is a tool with great potential for its use in different prevention programs and health promotions [11].

It is well known that the dawn of Web 2.0 resources has provoked a substantive change in the communication of knowledge, favouring its disclosure by enabling the expansion and permeability of knowledge at a very low cost. Regarding health education, it is necessary that the content system and messages related to prevention reach young people (the target of many job training programs) in the most informal and enjoyable way, for which the information and communication technologies would be of great utility [11]. Effective communication is necessary to achieve success.

Web 2.0 tools, especially Facebook, have shown positive effects in fostering prevention strategies and can contribute to attracting and engaging young people in health-related campaigns. These tools can be used in combination with other interventions. In either case, they have the potential to become indispensable public health tools [11]. In recent years, Facebook, YouTube, Twitter, and other websites have become effective tools for extending reach, promoting engagement, accelerating early detection, and increasing access to health messages [21,64,65].

In this same review, the work of Duregon et al. [66] endorsed the effectiveness of Facebook in promoting regular exercise and enhancing health indicators. Similarly, Jane et al. [67] supported the use of social networks to enable support and information sharing for the implementation of weight management programmes.

From the interventions observed, it was possible to deduce that the actions that included Web 2.0 tolls achieved adequate results in the working population. This statement is consistent with the results reported by Upadhyaya et al. [68], who concluded that occupational health professionals should continue to be creative in the development of multicomponent interventions. Likewise, Melian et al. [1] concluded that interventions carried out in the workplace, well planned and that included various strategies, were generally shown to be useful in combating overweight and obesity, which would reinforce what was observed in this review.

A correct diet, along with adequate hydration, has the potential to influence many aspects of work; however, nutritional interventions are scarce as a measure to improve the health and performance of workers [10].

### 4.3. Results of the Carried-Out Interventions

A significant decrease in the parameter under study, either BMI or BW, was observed in all but one of the trials analysed [39]. However, no relationship could be established between the four interventions used and the decrease in BMI or BW. This might be due to the heterogeneity of the methods and the criteria used. The diversity of studies based on Web 2.0 highlights the need to homogenise the methods and applications used. According to Lozano-Chacon et al. [69], this is a fundamental step for these platforms to be considered adequate tools for weight management in clinical practice. Also, according to Melián-Fleitas et al. [1], the results of this review showed that workplace interventions using Web 2.0 tools were effective in improving outcomes related to overweight and obesity.

Although it was not possible to ascertain which intervention was more effective, the use of Web 2.0 tools was found to help improve BMI or BW, albeit a larger body of evidence is needed to determine the strengths and limitations of this type of research and to convince health professionals [70]. Thus, policymakers should consider social entrepreneurs as partners in obesity prevention [5].

It highlights social media’s potential for weight loss interventions among younger groups. It is important to note that even small changes in behaviour, observed across entire populations, are likely to show significant effects on disease risk. The interpretation of the results of workplace intervention studies should be based on the public health significance of the outcomes or effects [3].

Although it has been mentioned that the results of these studies cannot be generalised to the general population, as shown by the results of the meta-analysis, this type of initiative is crucial because, as stated in the introduction, it must be emphasised that obesity in the population is a serious health problem that has not been tackled by any of the strategies used in the last 30 years [71]. Hence, the use of new tools is presented as a necessary alternative when nothing else has worked over the last 30 years [5].

Multiple studies have been carried out on occupational health. However, the role of other factors that may influence the health and productivity of workers, such as nutrition, is generally overlooked. For this reason, Shearer et al. [9] consider nutrition to be an integral part of a safe and productive workplace that encompasses physical and mental health as well as the long-term well-being of workers.

Having said that, information plays a fundamental role in healthy food and a balanced diet. However, despite its importance, there is little information on employees’ attitudes, behaviours, and information preferences, as well as vital details to targeted communication strategies. For this reason, it must ensure that commercial interests do not hamper occupational health policies and programs [12].

Infodemiology metrics follow population health-relevant events or predict them. Thus, these metrics and methods are potentially useful for public health practice and research and should be further developed and standardised [15]. Moreover, these metrics could be used in a more proactive way to assist public health officials in surveillance purposes [16]. The monitoring of online queries can provide insight into human behaviour, as this field is significantly and continuously growing and will be proven more valuable in the future for assessing behavioural changes and providing ground for research using data that could not have been accessed otherwise [17]. In addition, identifying trends and seasonal patterns in online interest through Web 2.0 tools can support resource allocation and planning and allows one to evaluate the effectiveness of global and local outreach efforts on public health [19]. Or even carry out more specific surveillance programs that increase the effective use of public resources [18].

In addition, all the extracted data provided by Infodemiology methodologies could be used with moderns Machine Learning algorithms to further improve traditional public health analysis methodologies [14].

Finally, we agree with Bragazzi et al. [20]: Web 2.0 tools could be useful to assess the reaction of the public and the level of public engagement both to novel risk factors associated with occupational diseases, and possibly related changes in disease natural history, and to the effectiveness of preventive workplace practices and legislative measures adopted to improve occupational health.

Further, occupational clinicians should become aware of the topics most frequently searched -on the Internet- by patients and proactively address these concerns during the medical examination. Institutional bodies and organisms should be more present and active in digital tools and media to disseminate and communicate scientifically accurate information. In any case, when using Web 2.0 tools in occupational health, it is crucial to prioritise data privacy and security, ensure that employees are adequately trained on using the tools, and consider any potential cultural or generational factors that might affect their adoption. Additionally, organisations should comply with relevant regulations and guidelines to protect employees’ personal health information.

### 4.4. Limitations

Certainly, there may be ‘technological biases’ (misinterpretation of available information) caused by the operation of Web 2.0, but these are impossible to control without access to the software tool. For this reason, there is growing concern that the use of artificial intelligence in health care may be disadvantageous to minority groups [72].

The main limitation of this systematic review is the originality of the methodologies used. This new aspect, both in the infodemiological part and in the use of Web 2.0 technologies for the interventions, has led to a high degree of heterogeneity in the value propositions presented by the articles, which makes it extremely difficult (as confirmed by the meta-analysis) to draw general conclusions from the joint analysis of the results.

Although the field is currently booming, there is less material available than in other areas of knowledge due to the short time that these techniques have been used in healthcare, and the scientific knowledge base is not yet well consolidated.

On the other hand, given the methodology used to select people for clinical trials, it was found that the greatest risk of bias in the trials stemmed from this stage of the process. In this regard, we have identified this as an area for improvement when using social networks for screening.

With all that in mind, there is logical to think that the meta-analysis would present some heterogeneity. The novelty of the subject, and the variability in the encountered approach to the study of Web 2.0 and Nutritional Disorders in conjunction, delivered a result that must be taken with care.

## 5. Conclusions

Despite the high heterogeneity of the results reported, the trend shown in all cases indicates that the intervention methodologies implemented by empowering individuals through Web 2.0 technologies are positive in terms of the problem of being overweight. Further implementation of novel strategies to support individuals is needed to overcome obesity, and, at least in the early studies, these strategies seem to be making the necessary change.

## Figures and Tables

**Table 3:** Adequacy of the studies using the 25 CONSORT guideline assessment items (only 24 are assessed, item 19 is not considered).

	1	2	3	4	5	6	7	8	9	10	11	12	13	14	15	16	17	18	19	20	21	22	23	24	25	Total	%
Hene et al., 2022 [31]	0.5	1	0.5	1	1	1	1	1	0	0	0	1	0.5	1	1	1	1	1	NA	1	1	1	0	0	1	17.5	72.9
Kariuki et al., 2022 [32]	0.5	1	1	1	1	1	1	1	1	1	0	1	1	0.5	1	1	1	0	NA	1	1	1	1	1	1	21.0	87.5
Napolitano et al., 2022 [33]	0.5	1	0.5	1	1	1	0.5	0.5	0	1	0	1	1	0.5	1	1	1	0	NA	1	1	1	0	0	1	16.5	68.8
Moholdt et al., 2021 [34]	1	1	1	1	1	1	1	1	1	1	1	1	1	1	1	1	1	1	NA	1	1	1	1	1	1	24.0	100.0
Biederman et al., 2021 [35]	0.5	1	0.5	0.5	1	0.5	0	0	0	0	0	1	0.5	0	1	1	1	1	NA	0	1	1	0	0	0	11.5	47.9
Xu et al., 2021 [36]	1	1	1	1	1	1	0	0	0	0	0	1	0.5	0	1	1	1	1	NA	1	1	1	1	0	1	16.5	68.8
Osborn et al., 2020 [37]	1	1	1	1	1	1	1	1	1	1	0.5	1	1	1	1	1	1	1	NA	1	1	1	1	1	1	23.5	97.9
Hawkins et al., 2020 [38]	0.5	1	1	1	1	1	1	1	1	1	0	1	0.5	1	1	1	1	0	NA	1	1	1	0	0	1	19.0	79.2
Dagan et al. 2015 [39]	0.5	1	0.5	0.5	1	0	0	0	0	0	0	0	0	1	1	1	1	1	NA	1	1	1	1	0	1	13.5	56.3
Huei Phing et al., 2015 [40]	0.5	1	1	1	1	1	0.5	1	1	0	0	0.5	1	1	1	1	1	1	NA	1	1	1	0	0	0	17.5	72.9
Merchant et al., 2014 [41]	0.5	1	1	1	1	0.5	0	1	1	0	0	0	0	1	1	1	1	1	NA	1	1	1	1	0	1	17.0	70.8

NA = Not apply.

## Data Availability

Not applicable.
